# The effects of microencapsulated hot chili pepper on the blood metabolites and physiological parameters of dairy cows

**DOI:** 10.14202/vetworld.2025.907-917

**Published:** 2025-04-23

**Authors:** Mónica Madrigal-Valverde, Marcus Vínicius Galvão Loiola, José Esler Freitas Junior, Murilo Ramos Santiago, Lara Lôbo Dantas, Artur Azevedo Menezes, Sandro Percário, Everton Luiz Pompeu Varela, Eduardo Costa, Endrigo Adonis Braga de Araujo, >Rodrigo Freitas Bittencourt

**Affiliations:** 1 Agronomy School, CTLSC, Tecnological Institute of Costa Rica (Instituto Tecnológico de Costa Rica) Costa Rica, 223-21001; 2 Área académica del Doctorado en Ciencias Naturales para el Desarrollo, Campus Tecnológico Local San Carlos, Instituto Tecnológico de Costa Rica, San Carlos, Costa Rica, 223-21001; 3 Doctorado en Ciencia Naturales de para el Desarrollo (DOCINADE), Instituto Tecnológico de Costa Rica, Universidad Nacional, Universidad Estatal a Distancia, Costa Rica; 4 School of Veterinary Medicine and Animal Science, Federal University of Bahia, Salvador, Brasil, 40170-110; 5 Programa de Pós-graduação em Biodiversidade e Biotecnologia da Rede BIONORTE, Brasil

**Keywords:** capsaicin, dairy cows, lipid metabolism, microencapsulated chili pepper, physiological responses, tropical livestock systems

## Abstract

**Background and Aim:**

The administration of hot chili pepper in cattle nutrition has been suggested to influence immune responses, antioxidant activities, and physiological parameters. This study aimed to evaluate the effects of microencapsulated hot chili pepper (MCP) supplementation on blood metabolites, antioxidant capacity, and physiological parameters in crossbred dairy cows, contributing novel insights into metabolic and physiological adaptations under tropical production systems.

**Materials and Methods:**

Twenty-four crossbred lactating cows (*Bos taurus* × *Bos indicus*); average body weight 447.8 ± 89.6 kg; 53.54 ± 11.8 days in milk; average daily milk production 6.34 ± 1.91 L/cow/day) were randomly divided into two groups: A control group without additives and a treatment group supplemented with MCP (1,000 mg/cow/day) for 42 days. Blood samples were collected weekly for hematological analysis, biochemical evaluations, and oxidative stress biomarkers (2,2-diphenyl-1-picrylhydrazyl [DPPH], thiobarbituric acid reactive substances [TBARS], and trolox equivalent antioxidant capacity [TEAC]). Physiological measurements, including body condition score, heart rate, respiratory frequency, and urine pH, were also assessed weekly. Data were statistically analyzed using mixed-model procedures with repeated measures over time.

**Results:**

Significant interactions between treatment and time were observed for hematocrit, red blood cells, and hemoglobin (p < 0.05), indicating physiological adaptations potentially related to increased water intake. Serum albumin levels were significantly lower in MCP-supplemented cows compared to controls (p = 0.006), suggesting a modulation of lipid transport mechanisms. Temporal variations significantly affected 75% of hematological parameters and 83% of biochemical parameters. Although antioxidant parameters (DPPH, TBARS, TEAC) did not differ significantly between groups, notable temporal changes were observed (p < 0.05). Physiological parameters showed significant temporal variations but no consistent effects due to MCP supplementation.

**Conclusion:**

Supplementation of dairy cows with MCP significantly impacted hematological parameters and serum albumin levels, revealing potential metabolic adjustments involving lipid transport and hydration status. However, oxidative stress markers and physiological parameters remained largely unaffected by the supplementation. These findings support the potential regulatory role of MCP in dairy cow metabolism, emphasizing its relevance as a dietary additive in tropical livestock production systems.

## INTRODUCTION

The postpartum period in dairy cows is characterized by reduced immune function, leading to health disorders and loss of pregnancy [1, 2]. These health disorders are associated with reproductive performance. In addition, reduced fertility and pregnancy loss are linked to blood profiles [1, 3]. The metabolism of dairy cows is intricately regulated to maintain homeostasis, but it is highly sensitive to disturbances caused by the overproduction of reactive oxygen species (ROS). These molecules, which are produced during normal metabolic processes, can accumulate under stress conditions, leading to oxidative stress [4–7]. In particular, oxidative stress has been linked to embryonic DNA lesions, uterine epithelial damage, endometritis, and abnormal reproductive development, which may contribute to infertility, pregnancy loss, and reduced efficiency of *in vitro* embryo production [4–6].

To mitigate these adverse effects, antioxidant compounds have been studied as potential dietary supplements in animal production systems. Polyphenols, for example, interact with ruminal microorganisms and influence fermentation patterns, protein degradation, and lipid metabolism [8, 9]. Among natural antioxidant sources, hot chili peppers are particularly notable for their high levels of phenolic compounds, flavonoids, and capsaicin – a bioactive compound with demonstrated immunomodulatory properties [10]. Capsaicin has the potential to enhance bovine immunity by increasing the immune response [11]. In addition, supplementation with chili-derived extracts has been associated with improved thermoregulation in cattle, as evidenced by lower rectal temperatures, which are particularly relevant in tropical environments [12, 13]. The metabolic effects of such supplementation have been demonstrated in primiparous dairy cows that have been administered such supplementation exhibit a reduction in serum insulin concentrations and a quadratic increase in β-hydroxybutyrate levels, which are markers of energy metabolism [11, 12]. These findings indicate that capsaicin has potential as an antioxidant and metabolic modulator in dairy cows.

Despite the established immunomodulatory and antioxidant properties of capsaicin derived from hot chili peppers, limited research exists regarding its influence on blood metabolites, physiological parameters, and oxidative stress responses, specifically in dairy cows under tropical production conditions. The use of microencapsulated forms of hot chili pepper as a dietary additive, which could potentially enhance bioavailability and prolong the bioactive effects of capsaicin, remains largely unexplored in bovine nutritional studies.

This study aims to investigate the effects of dietary supplementation with microencapsulated hot chili pepper (MCP) on blood metabolites, antioxidant activity, and physiological parameters in crossbred dairy cows under tropical environmental conditions. The outcomes of this research are intended to provide novel insights into the metabolic and physiological adaptations induced by capsaicin supplementation, potentially contributing to the development of improved nutritional strategies to enhance dairy cow health and productivity.

## MATERIALS AND METHODS

### Ethical approval

The animal study protocol was approved by the Committee for Ethics in the Use of Animals (CEUA) (CEUA, Abbreviation in Portuguese) of the Federal University of Bahia (protocol number 29/2022).

### Study period and location

The study was conducted from September to November 2022 (42 days). The experimental work was conducted at Fazenda Experimental Entre Rios, a property of the School of Veterinary Medicine and Animal Science at the Federal University of Bahia (EMVZ-UFBA), located in Entre Ríos, Bahia, Brazil (11°56’31”S38°05’04”W). The farm’s forage cover consists of *Brachiaria decumbens*, with an animal density of 1.2 animal units/ha. The average daily temperature was 25.75°C ± 1.35°C and the average daily humidity was 85.42% ± 5.61%.

### Animals

The experiment involved 24 crossbred lactating females (*Bos taurus* × *Bos indicus*) with 53.54 ± 11.8 days in milk, an average age of 7.16 ± 3.41 years, an average body weight (BW) of 447.80 ± 89.6 kg, and an average daily milk production of 6.34 ± 1.91 L/cow/day. Group assignment was performed using the Diman (Dimensional Analysis Software, Diman Sp, z.o.o, Poland). Table 1 [14, 15] shows the dry matter (DM) intake, BW, body score condition, and daily milk production for each group.

**Table 1:** Statistical mean for different treatments of crossbred cows supplemented or not with chili pepper microencapsulated.

Item	Treatment^1^		EPM^2^	p-value^3^		
	
	CT	CP		Treatment	Week	Inter
DMI (kg/d)^3^	11.98	12.39	0.77	0.139	0.0002	0.0002
BCS (1–5)^4,5^	2.53	2.31	0.40	0.048	< 0.0001	0.569
BW (kg)^4,5^	453.1	430.1	74.91	0.373	0.039	0.151
MY (kg/d)^6^	8.29	9.59	2.52	0.144	< 0.0001	0.011

1 Experimental Treatment, CT=Control treatment, CP=Chili pepper treatment;

2 EPM: standard error of the mean

3 Probability effect for Treatment. Week and interaction between Treatment and week (Inter); ^3^Results in an unpublished study Madrigal-Valverde *et al*. [14];

4 Results explain in discussion section;

5 Statistically homogeneous groups at the beginning of the experiment;

6 Results published in Madrigal-Valverde *et al*. [15]; BCS=Body condition score, BW=Body weight, MY=Milk yield, DMI=Dry matter intake

### Experimental design and feed analysis

This study employed an unrestricted randomized design in which animals were randomly assigned to one of two supplementation groups. Both groups received balanced feed, silage, and equal quantities of food. The control group (Control treatment [CT], n = 12) received balanced feed without microencapsulated chili pepper, whereas the treatment group (Chili pepper treatment [CP], n = 12) received concentrated feed with 1 g/cow/day of the Capcin^®^ product (to enhance absorption and sustain capsaicin’s effects over time, a novel approach in ruminant nutrition). The feed additive contains 5 g/kg capsaicinoids.

For the bromatological analysis, the samples of forage, ingredients, and reject material were dried in a convection oven with forced air (at 55°C for 72 h), and the samples were then ground through a 1-mm sieve. For the analysis of DM ash, crude protein, ethereal extract, acid detergent fiber, and lignin of forage, silage, and concentrate. The analyses were performed under the Association of Official Analytical Chemists international standards [16]. In turn, the samples were analyzed for neutral detergent fiber according to the methods of Mertens [17], using a thermostable alpha-amylase and sodium sulfite. Non-fibrous carbohydrates were calculated using the equation proposed by Hall [18]. The total digestible nutrients were calculated according to the NRC [19], and net energy for lactation was calculated as described by Weiss *et al*. [20]. The indigestible crude protein values were discounted from the DM consumption. Table 2 [11, 19] lists the chemical and bromatological composition of the ingredients used in the diets.

**Table 2:** Chemical and bromatological composition of ingredients used in experimental diets of all animals based on dry matter.

Item (% DM)	Supplement	Grass Pangola (*Digitaria decumbens*)	Corn Silage
Ground corn	60.00	-	-
Soybean meal	34.00	-	-
Urea	3.20	-	-
Mineral^1^	2.80	-	-
Chemical composition (% DM)^2^			
DM	93.86	88.97	36.36
OM	93.7	91.89	94.97
Ash	6.30	8.11	5.03
CP	25.39	5.60	8.10
EE	4.00	5.50	4.97
NDF	10.95	70.95	47.12
ADF	6.97	39.66	24.81
Non-fiber carbohydrates^3^	53.36	9.84	34.78
Total digestible nutrients (g/kg)^4^	582.46	64.76	73.44
Net energy (Mcal/kg of DM)^5^	7.46	1.47	1.68

1 Contained per kilogram of product: 225 g calcium, 160 g phosphorus, 30 g sulfur, 18 g magnesium, 120 mg cobalt, 2,500 mg copper, 120 mg iodine, 1,800 mg manganese, 36 mg selenium, 5,250 mg zinc, 1,600 mg fluorine.

2 DM=Dry matter, OM=Organic matter, CP=Crude protein, EE=Ether extract

3 According to Hall [18]:

4 According to the NASEM [19] equation.

5 According to the NASEM [19]. animal unit (standardized to a relative BW of 450 kg).

^6^In the CP group 1 g of capcin/cow/day was included

### Blood collection

Blood samples were collected weekly, beginning on the 1^st^ day of supplementation and ending on the 42^nd^ day. In the case of biochemical and oxidative stress blood analyses: The blood was collected and separated serum samples, as described by Madrigal-Valverde *et al*. [15]. For hemogram analysis, the same method was used, except that 2 mL tubes without anticoagulant were used (Vacutainer, Becton, Dickinson and Company, Franklin Lakes, New Jersey, United States). After collection, samples were stored in a cooler at 4°C and subsequently transported to the Clinical Analysis Laboratory at EMVZ-UFBA.

### Blood parameters

In the hemogram, the quantified parameters were hemoglobin (HGB), hematocrit (HCT), mean corpuscular volume (MCV), mean corpuscular hemoglobin (MCH), MCH concentration (MCHC), red cell width distribution, total leukocytes, segmented neutrophils, lymphocytes, monocytes, eosinophils, basophils, and platelets.

In the case of blood biochemistry, the variables of serum albumin, total cholesterol, triglycerides, high- density lipoproteins (HDL), very low-density lipoproteins (VLDL), low-density lipoproteins (LDL), and glucose were evaluated. Serum albumin was analyzed using the colorimetric method (Green-Bromocresol) and lipid profiles were analyzed using the enzymatic colorimetric method.

The metabolites analyzed to estimate oxidative stress were 2,2-diphenyl-1-picrylhydrazyl (DPPH), thiobarbituric acid reactive substances (TBARS), and trolox equivalent antioxidant capacity (TEAC) according to the methodologies described by previous studies [21–27]. After blood collection, the samples were placed in a horizontal position at room temperature for 50 min. After 50 min, the tubes containing blood were placed in a centrifuge (Daiki 80 2B, Brazil), where they were processed at a speed of 1,500× *g* for a period of 15 min.

### Physiological measurement

All measurements were collected weekly starting on the 5^th^ day of supplementation by the same technician. Each animal was taken to a scale to obtain the animal’s BW. The BCS was measured on a scale of 1–5, according to the method described by Singh *et al*. [26] and Jo Heart rate and respiration were measured before supplementation. A urine sample was collected in a clean bottle during each urination process. Immediately after collection, a pH meter was placed in the bottle (ChesseLab, Apera) to estimate the pH.

### Statistical analysis

The data were analyzed using the PROC MIXED procedure of Statistical Analysis System (SAS) (SAS for Windows 9.4, SAS Institute Inc., Cary, USA) according to the model of repeated measures over time, with the normality of residues and homogeneity of variances verified by the PROC UNIVARIATE procedure. This study introduces a longitudinal, time-dependent approach to analyze the interactions between capsaicin supplementation and metabolic changes over a 42-day experimental period, providing a more dynamic perspective than previous static evaluations. Repeated measurements were performed using the SAS MIXED procedure to analyze the week (1, 2, 3, 4, 5, 6, and 7 after supplementation), according to the following general model:

Y_ijk_ = µ + A_k_ + D_i_ + T_j_ + D_i_ XT_j_ + e_ijk_

Where Y_ijk_ is the dependent variable; µ the general average; A_k_ is the random effect of the animal; Di is the fixed effect of the diet; T_j_ is the fixed effect of time (weeks); D_i_XT_j_ is the interaction effect of diet and time; and e_ijk_ is the residual error.

The degrees of freedom were calculated according to the Satterthwaite method (ddfm = satterth). Self-regression 1 obtained the best covariance structure based on the lowest Akaike information criterion values. Other covariance structures were tested, including composite symmetry, heterogeneous composite symmetry, and unstructured and heterogeneous autoregression. All variables collected after supplementation were individually subjected to the F test using the least-square means. Significance was set at p < 0.05. All animal parameters that could alter the results were included in the statistical model as covariables, such as animal weight, lactation duration, body score, and milk yield.

## RESULTS

### Blood parameters

This study presents new evidence of significant interactions between dietary capsaicin supplementation and hematological parameters over time, suggesting a dynamic physiological response rather than a simple linear effect. A trend toward statistical significance was observed for both HCT and red blood cell (RBC) counts (Table 3). Furthermore, nine parameters exhibited significant differences based on the time criterion (p ≤ 0.05), with 61.1% of the blood dynamic parameters being influenced by temporal changes. In addition, parameters such as HGB (g/dL) and eosinophil counts (µL) exhibited a statistical trend (p < 0.10), suggesting potential physiological adaptations over time.

**Table 3:** Statistical mean blood metabolite parameters for the different treatments of crossbred cows supplemented or not with chili pepper microencapsulated.

Item	Treatment^1^		EPM^2^	p-value^2^		
	
	CT	CP		Treatment	Week	Inter
Basophils (%)	0.10	0.15	0.38	0.462	0.556	0.802
Basophils (µL)	10.09	20.88	58.18	0.287	0.552	0.378
Eosinophils (%)	14.45	11.01	10.15	0.194	<0.0001	0.505
Eosinophils (µL)	1338.91	1018.30	1042.34	0.308	<0.0001	0.096
Hemoglobin (g/dL)	10.24	9.95	1.44	0.433	<0.0001	0.059
Hematocrit (%)	30.54	29.06	3.99	0.22	0.002	0.010
Lymphocytes (%)	50.97	50.64	15.57	0.927	0.1201	0.111
Lymphocytes (µL)	4529.02	4433.26	1970.03	0.831	0.025	0.101
Monocytes (%)	176.30	163.78	187.32	0.739	<0.0001	0.289
Monocytes (µL)	5.90	7.26	35.04	0.848	0.47	0.313
MCHC (g/dL)	33.58	34.29	2.92	0.186	<0.0001	0.254
MCV (fL)	59.99	59.16	8.34	0.92	<0.0001	0.179
Neutrophils (%)	2818.20	2841.28	2018.76	0.955	0.038	0.765
Neutrophils (µL)	30.15	32.10	14.78	0.55	<0.0001	0.937
PLT (×109/L)	244.47	229.31	140.03	0.628	0.007	0.248
Red blood cell (×10^12^/L)	5.11	4.93	0.56	0.234	<0.0001	0.022
WBC (/µL)	8922.20	8765.52	2995.08	0.808	0.006	0.524
Albumin (g/dL)	3.26	3.10	0.27	0.006	<0.0001	0.043
Glucose (mg/dL)	47.50	47.80	17.10	0.918	<0.0001	0.651
HDL (mg/dL)	52.76	47.20	11.73	0.077	<0.0001	0.515
LDL (mg/dL)	92.32	84.30	24.96	0.334	<0.0001	0.315
Total cholesterol level (mg/dL)	149.70	135.48	32.54	0.221	<0.0001	0.260
Triglycerides (mg/dL)	23.02	19.92	17.10	0.343	0.115	0.355
VLDL (mg/dL)	4.60	3.98	1.22	0.343	0.115	0.353
DPPH (mM/L)	0.32	0.31	0.01	0.81	<0.0001	0.10
TBARS (µM/L)	1.18	1.14	0.04	0.18	< 0.0001	0.14
TEAC (mM/L)	1.61	1.60	0.03	0.68	< 0.0001	0.15

1 Experimental treatment, CT=Control treatment, CP=Chili pepper treatment,

2 EPM: standard error of the mean,

^3^Probability effect for Treatment. Week and interaction between treatment and week (Inter). MCV=Mean corpuscular volume, MCHC=Mean corpuscular hemoglobin concentration, PTL=Platelet count test, WBC=Number of white blood cells, Neutrophils=Absolute neutrophils count/µL, Lymphocytes=Absolute lymphocytes count/µL, Eosinophils=Absolute eosinophils count/µL, Basophils=Absolute basophils count/µL, Monocytes Mono=Absolute monocytes count/µL, HDL=High-density lipoprotein, LDL=Low-density lipoprotein, VLDL=Very low-density lipoprotein, DPPH=2,2-Difenil-1-picrilhidrazil, TBARS=Thiobarbituric acid reactive substances, TEAC=Trolox equivalent antioxidant capacity

**Figure 1:**
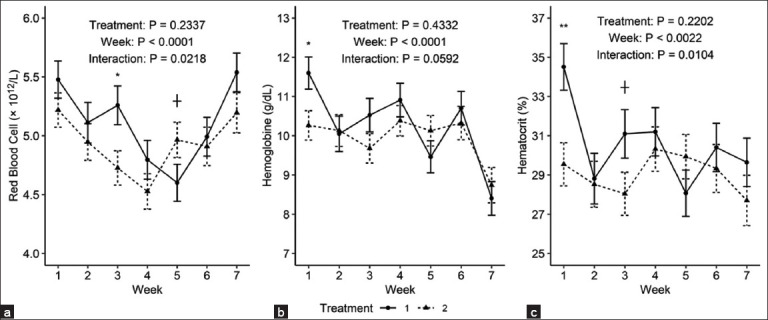
(a) Hemoglobin (g/dL), (b) Hematocrit (%), and (c) red blood cells (×10^12^/L) in dairy cows postpartum between experimental groups 1: Control treatment, 2: Chili pepper treatment.

Regarding weekly blood parameter variations, (HGB, g/dL) and (HCT, %) exhibited fluctuations over time (Figure 1).

Significant week-to-week differences (p < 0.05) were observed in several parameters, including (RBC, × 10¹²/L), (HGB, g/dL), (HCT, %), (MCV, fL), (MCHC, g/dL), neutrophils (µL), lymphocytes (µL), eosinophils (µL), basophils (µL), and monocytes (µL) (Table 2) [11].

For blood metabolites evaluated through biochemistry analysis, albumin (g/dL) exhibited significant differences based on group, week, and group-week interaction (p = 0.006) (Table 2) [11].

Other parameters showed no significant differences (p ≤ 0.05) in the evaluated parameters for the time group interactions and for the groups in the evaluated parameters. On the other hand, for the time criterion, there were significant differences (p ≤ 0.05) for 83% of the parameters evaluated.

The weekly analysis of HDL (mg/dL) indicated a trend toward a significant difference between the treatment and control groups (p < 0.05) in week 2 (p = 0.054), week 6 (p = 0.064), and week 7 (p = 0.059) (Table 3).

On the other hand, no notable discrepancies were identified with regard to oxidative stress parameters in relation to the treatment time interaction or treatment effects in isolation (p ≥ 0.05). Nevertheless, for all three parameters, a significant difference was identified with regard to the time variable (p < 0.05). Table 3 presents a detailed analysis of the observed behavior of the parameters over the 7-week experimental period.

**Figure 2:**
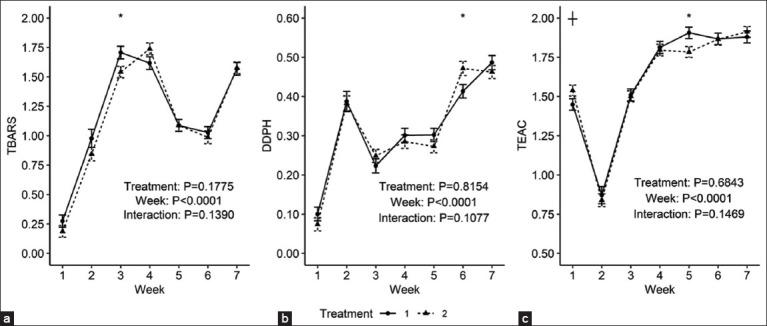
(a) 2,2-Difenil-1-Picrilhidrazil, (b) Thiobarbituric acid reactive substances, and (c) Trolox equivalent antioxidant capacity in dairy cows postpartum between experimental groups 1: Control treatment, 2: Chili pepper treatment.

In week 6, the CP group exhibited a significantly higher value for the DPPH (mM/L) parameter than the CT group (0.47 vs. 0.41, p < 0.81). Regarding the TBARS (µM/L) parameter in week 3, a statistically significant difference was observed between the treatments (p < 0.18), with the CP group exhibiting a lower value (1.54 vs. 1.71). A significant difference was observed at the outset of the experiment for the TEAC (mM/L) parameter, with the CP group exhibiting a higher value (1.54 vs. 1.45, p < 0.68). However, this parameter demonstrated a decline in the CP group by week 5 (1.78 vs. 1.91, p = 0.02) (Figure 2).

### Physiological parameters

Regarding the behavior of the physiological parameters, there were no significant differences (p ≥ 0.05) for the interaction between treatment and time or for the treatment. For the time variable, significant differences (p < 0.05) were reported in the BCS (1–5), BW (kg), cardiac frequency (beats per minute), and respiratory frequency (breaths per minute) parameters (Table 4).

**Table 4:** Statistical mean physical and physiological parameters for the different treatments of crossbred cows supplemented or not with chili pepper microencapsulated.

Item	Treatment^1^		EPM^2^	p-value^2^		
	
	CT	CP		Group	Week	Inter
CF (BPM)	77.87	74.74	13.89	0.256	<0.0001	0.661
RF (BRPM)	38.56	41.83	14.23	0.336	<0.0001	0.390
RT (°C)	38.62	38.72	0.47	0.406	0.313	0.640
PhUrine (1–14)	7.98	7.87	0.49	0.010	<0.0001	0.799

1 Experimental treatment, CT=Control treatment, CP=Chili pepper treatment;

2 EPM: standard error of the mean;

^3^Probability effect for treatment. Week and interaction between treatment and week (Inter). BCS=Body condition score, CF=Cardiac frequency (beats per minute), RF=Respiratory frequency (breaths per minute), RT=Rectal temperature

The body condition of the animals in both groups ranged from 2.1 to 2.8 on a scale of 1–5. For week 6, a significant difference (p = 0.04) can be observed between the treatments (2.25 CT, 2.21 CP).

The heart and respiratory rates of the animals in the control group and those supplemented with microencapsulated hot chili did not show significant differences (p < 0.05). The respiratory rate ranged from 25 to 50 breaths/min, while the heart rate ranged from 65 to 85 beats/min (Table 4). In week 3, significant differences (p < 0.05) were recorded between treatments for the respiratory rate (39.63 CT, 49.92 CP, BRPM) in week 3 and the heart rate in week 4 (85.09 CT, 74.50 CP, BPM).

## DISCUSSION

The peripartum and early lactation periods in dairy cows are marked by physiological stress, which can affect inflammatory responses and immune function [24, 28]. This study aimed to evaluate whether MCP supplementation can alleviate stress responses and support immune function during early lactation.

We monitored a range of hematological parameters, including basophils, eosinophils, HGB, HCT, MCHCs, MCVs, PLTs, RBCs, lymphocytes, monocytes, neutrophils, and white blood cells (WBCs). The values for HGB, HCT, MCHCs, MCVs, PLTs, and RBCs remained within the normal ranges for dairy cows [29, 30]. Basophil percentages were consistent with those reported in previous studies in which cows were supplemented with monensin or monensin combined with capsicum at a dose of 250 mg/day. However, the eosinophil percentages were elevated in both groups, which is consistent with the findings of similar studies.

In the blood count, the lymphocyte (3820 ± 420 cells/µL) and monocyte (790 ± 39 cels/µL) counts exceeded the parameters observed in the control group, whereas the WBC counts (8400 ± 0.65) were similar to those reported for the control groups in dairy cow studies [31, 32]. It is notable that lymphocyte levels can be influenced in response to both immune challenges and dietary alterations [33, 34]. These observations suggest that capsaicin supplementation supports immune function without compromising overall health, highlighting its potential as a functional feed additive in dairy.

Lymphocytes, monocytes, and WBCs had values similar to those reported in the studies by Silva *et al*. [32] and Bertoni *et al*. [31]. In addition, the weekly values of neutrophils, lymphocytes, and WBCs were below or similar to those reported for healthy multiparous cows by Piñeiro *et al*. [35] and Yang *et al*. [36], who found intervals of 3–3.5 × 10^6^ mL for neutrophils, 4.1–4.8 × 10^6^ mL for lymphocytes, and 7.4–9.4 × 10^6^ mL for WBCs. These results indicate that the animals studied were healthy.

Weekly analysis of blood count parameters revealed that variations were within the normal range. These variations reflected natural immune system fluctuations and were not attributable to capsaicin treatment.

The blood count results showed that the percentage of lymphocytes (%) was similar to that reported by Westphalen *et al*. [13] (49.57%–50.55%). In their study, capsaicin levels were similar between the control group and the groups supplemented with both 250 and 500 mg/d of capsaicin (41.6, 41, and 42.3, respectively). The levels of basophils were similar (500 mg/d = 0.17 and control 0.15), and red cells were between 5 and 6 × 10^12^/L in groups of cattle that were offered 250, 500, and 1,000 mg/d (5.70, 5.85, and 5.66, respectively) [11] and the control group (5.66 mg/d). All of the abovementioned parameters are comparable with those in our study. This suggests that capsaicin does not have a significant effect on lymphocyte counts.

On the other hand, for the HGB parameter in animals supplemented with capsaicin, the levels were lower in this study than in Westphalen *et al*. [13], and Oh *et al*. [11] found significant differences (p < 0.05) in the amount of HGB between groups supplemented with capsaicin and their control group, which increased according to the dosage of capsaicin [11]. Nevertheless, lower HGB levels were reported in the groups supplemented with capsaicin (12.16 g/dL) compared with the control group (12.65 g/dL) (p < 0.05) [13]. This indicates that capsaicin does not significantly alter RBC production.

Differences (p < 0.05) in the treatment group interaction were observed in HCT (%), erythrocytes (×10^12^/L), and the trend in HGB (g/dL) levels. The slightly lower HCT, RBCs, and HGB levels in groups of animals with identical environmental and health conditions may indicate an effect of increased plasma levels due to water consumption (hemodilution). Hypothesis exist that capsaicin increases water intake because capsaicin compounds influence deglutition via the vagus nerve and activate transient receptor potential vanilloid member 1 in the tongue and duodenum [37, 38]. In another study by Jo *et al*. [29] and Martínez Marín *et al*. [30], the group of animals with the highest water consumption had lower HGB levels.

In contrast, the biochemical parameters albumin, glucose, HDL, LDL, total cholesterol, triglycerides, and VLDL were considered. The glucose parameter in our study was below that reported by Oh *et al*. [28] and Araujo *et al*. [39] (126 and 127 mg/dL) in animals supplemented with 100 and 200 mg/d of capsicum. However, in the indicated study, the animals were not grazed; instead, fibrous supplies were provided with hay and corn silage. Therefore, the difference in glucose levels between our study and other studies could be attributable to variations in diet, management, or other factors. However, in animals that did not consume capsaicin in our study, the glucose values were in the range reported by Jo *et al*. [29] and Martínez Marín *et al*. [30].

This study provides novel insights into the relationship between capsaicin supplementation and albumin metabolism (p < 0.05), suggesting a potential regulatory role of capsaicin in lipid transport mechanisms, which warrants further investigation. The group supplemented with capsaicin (1 g/d) presented lower levels of serum albumin than the control group. In animals that did not consume capsaicin, albumin values were in the range reported in Jo *et al*. [29] and Martínez Marín *et al*. [30].

The capsaicin group had lower albumin levels, which could be associated with lower level of short-chain fatty acid and cholesterol level. Albumin transports fatty acids of <12 carbons, such as non-esterified acids, through the portal system. The availability of non-esterified fatty acids is important during the postpartum period and early lactation, where they are made available to the mammary glands [30, 40]. The fatty acids transported by albumin include cholesterol derivatives such as hormones, which may explain the positive correlation (r = 0.995) between cholesterol levels and serum albumin [41, 42]. In turn, serum albumin is an indicator of liver function, and its response is inflammatory [31, 34, 43, 44]. High neutrophil, eosinophil, and basophil counts are related to low levels of albumin/globulin [34, 44].

The lower albumin levels in the capsaicin-supplemented group suggested reduced short-chain fatty acid and cholesterol levels, potentially reflecting the beneficial effects of capsaicin [36, 45]. Lower albumin levels may be related to the anti-inflammatory properties of capsaicin [36, 45]. The albumin levels in both groups were within the normal range for crossbred cattle [39, 46]. This suggests that the difference in albumin levels between the groups was relatively small and may not have significant clinical implications.

The results indicate that LDL, triglycerides, and VLDL levels were similar to the parameters reported for healthy cows from the same farm [39, 46]. However, the total cholesterol and HDL levels were lower than those in the studies by Araujo *et al*. [39] and Vittorazi *et al*. [46]. This is associated with decreased albumin levels. Unlike previous studies that only examined metabolic changes, this study incorporates oxidative stress biomarkers (DPPH, TEAC, TBARS) to provide a more comprehensive understanding of capsaicin’s impact on dairy cow physiology. It is important to point out that there is a paucity of research on oxidative stress markers in the blood of dairy cows during early lactation, which makes this investigation particularly relevant.

It has been demonstrated that capsaicin exerts a beneficial effect on cardiovascular health in animals by reducing oxidative stress [36, 45]. The antioxidant properties of DPPH are ascribed to its capacity to scavenge free radicals, as evidenced by laboratory studies employing DPPH assays. Furthermore, capsaicin has been evaluated for its antimicrobial activity against a range of bacterial pathogens, including *Staphylococcus aureus*, *Salmonella enterica*, and *Escherichia coli* [40, 47]. The antioxidant effect of the compound is linked to its interaction with cytochrome c in mitochondria, which helps regulate cellular apoptosis and prevents excessive ROS production. Elevated ROS levels have been linked to cellular and tissue damage and intrinsic cellular apoptosis [41, 43, 48].

Despite the confirmed antioxidant capacity of capsaicin *in vitro*, this study did not identify any significant differences in oxidative stress parameters between the control and capsaicin-supplemented groups. These findings are in accordance with those of Oh *et al*. [11], who reported no significant differences (p > 0.05) in TBARS activity between the control and capsaicin-supplemented groups (250, 500, and 1,000 mg/d). The antioxidant effects of capsaicin observed *in vitro* may not directly translate to quantifiable changes in the blood of supplemented animals under the conditions studied.

The body condition findings in our study are consistent with those of lactating animals in the early lactation stage. Body condition declined from the 4^th^ week of treatment, which was justified by the increase in energy necessary for animals to reach their peak lactation. There were no significant differences in body condition between the two groups. Similarly, in a study in which dairy cows were supplemented with yeast, the control and supplemented groups maintained similar body conditions throughout the experiment [44].

In other studies, neither BW nor body condition differed between the control group and the groups that consumed bioactive compounds [45] or botanical mixtures [12]. In animals that consumed capsaicin, no differences were found between the control and supplemented groups (15 mg/kg/DM/d of capsaicin) (p ≥ 0.05) in the initial BW (p = 0.99) or final weight toward the end of the experiment (p = 42) [13].

Vittorazi *et al*. [46] study evidences that there were no significant differences in the use of a microencapsulated hot chili extract compound (75 and 150 mg/d [Capsin^®^]) (p < 0.05) in BW. However, they observed a time effect, as observed in this study. The recorded respiratory frequency exceeded the normal range for adult bovines (12–36 breaths/min); however, the heart rate remained within the expected range for adult cattle (60–80 BPM) [47]. The unreported effect of capsaicin on thermoregulation in dairy cows is an important finding given the increasing need for climate-adaptive feeding strategies. The respiratory frequency did not show significant differences (p ≥ 0.05) between groups of dairy cows that consumed 75 or 150 mg/d of capsaicin (Capcin^®^). However, a significant difference was observed for the time variable (p < 0.05), which reaffirms the results of our study by Vittorazi *et al*. [46]. This study also observed the same heart rate behavior reported in this study by Vittorazi *et al*. [46].

In another study by Banuelos and Stevenson [48], the rectal temperatures of animals with a condition of 2–3 were 38.96 ± 0.04 and 38.87 ± 0.04, which are higher than those reported in this study. However, researchers have indicated that groups with higher temperatures had a higher incidence of low postpartum and early lactation ovulation, with the reported temperatures being favorable for the reproduction of dairy cows.

## CONCLUSION

This study demonstrated that dietary supplementation of crossbred dairy cows with MCP at a dosage of 1 g/cow/day significantly influenced specific blood metabolites, notably resulting in lower serum albumin levels compared to the control group, suggesting potential modulation of lipid transport mechanisms. Significant temporal interactions were observed in hematological parameters such as HCT, HGB, and RBC counts, indicating possible metabolic adaptations related to hydration status or altered water consumption behavior. However, MCP supplementation did not substantially affect oxidative stress biomarkers (DPPH, TBARS, and TEAC) or physiological parameters, indicating a limited effect of this supplementation on systemic antioxidant capacity and thermoregulatory responses under the conditions studied.

The robustness of this research lies in its longitudinal approach, enabling the assessment of dynamic interactions between MCP supplementation and physiological and metabolic responses over time. In addition, the use of microencapsulated capsaicin represents an innovative strategy aimed at improving compound stability, bioavailability, and sustained release, a significant advancement in ruminant nutritional supplementation strategies.

One limitation is the relatively short duration (42 days) and small sample size (n = 24), which may have restricted the detection of subtle long-term effects or interactions. Furthermore, the study was limited to tropical conditions, potentially restricting the generalizability of findings to other climatic or management systems. The absence of direct measurements of water intake and rumen metabolism may also constrain the interpretation of certain physiological adaptations observed in this study.

Further research should explore extended supplementation periods and larger sample sizes to validate and expand upon these findings. Investigations incorporating direct measurements of water and feed intake, rumen microbial dynamics, and detailed lipid metabolism pathways could provide deeper insights into the mechanisms underlying the observed effects of capsaicin supplementation. In addition, exploring the effects of varying MCP doses and its impacts under different environmental stress conditions could help optimize its application as a nutritional strategy in diverse cattle production systems.

## AUTHORS’ CONTRIBUTIONS

JEFJ and MCGL: Conceptualization. MMV, AAM, LLD and MRS: Methodology. JEFJ and MMV: Formal analysis. SP, ELPV, MMV, and EC: Investigation. RFB: Resources. MMV: Data curation. MMV: Writing - original draft preparation. JEFJ, MVGL, and EABA: Writing - reviewed and edited the manuscript. EABA, JEFJ, and MVGL: Supervised the manuscript. All authors have read and agreed to the published version of the manuscript.
